# Synaptic Inhibition of Medial Olivocochlear Efferent Neurons by Neurons of the Medial Nucleus of the Trapezoid Body

**DOI:** 10.1523/JNEUROSCI.1288-19.2019

**Published:** 2020-01-15

**Authors:** Lester Torres Cadenas, Matthew J. Fischl, Catherine J.C. Weisz

**Affiliations:** Section on Neuronal Circuitry, National Institute on Deafness and Other Communication Disorders, NIH, Bethesda, Maryland 20892

**Keywords:** auditory, efferent, inhibitory synapse, medial olivocochlear, MNTB, OHC

## Abstract

Medial olivocochlear (MOC) efferent neurons in the brainstem comprise the final stage of descending control of the mammalian peripheral auditory system through axon projections to the cochlea. MOC activity adjusts cochlear gain and frequency tuning, and protects the ear from acoustic trauma. The neuronal pathways that activate and modulate the MOC somata in the brainstem to drive these cochlear effects are poorly understood. Evidence suggests that MOC neurons are primarily excited by sound stimuli in a three-neuron activation loop from the auditory nerve via an intermediate neuron in the cochlear nucleus. Anatomical studies suggest that MOC neurons receive diverse synaptic inputs, but the functional effect of additional synaptic influences on MOC neuron responses is unknown. Here we use patch-clamp electrophysiological recordings from identified MOC neurons in brainstem slices from mice of either sex to demonstrate that in addition to excitatory glutamatergic synapses, MOC neurons receive inhibitory GABAergic and glycinergic synaptic inputs. These synapses are activated by electrical stimulation of axons near the medial nucleus of the trapezoid body (MNTB). Focal glutamate uncaging confirms MNTB neurons as a source of inhibitory synapses onto MOC neurons. MNTB neurons inhibit MOC action potentials, but this effect depresses with repeat activation. This work identifies a new pathway of connectivity between brainstem auditory neurons and indicates that MOC neurons are both excited and inhibited by sound stimuli received at the same ear. The pathway depression suggests that the effect of MNTB inhibition of MOC neurons diminishes over the course of a sustained sound.

**SIGNIFICANCE STATEMENT** Medial olivocochlear (MOC) neurons are the final stage of descending control of the mammalian auditory system and exert influence on cochlear mechanics to modulate perception of acoustic stimuli. The brainstem pathways that drive MOC function are poorly understood. Here we show for the first time that MOC neurons are inhibited by neurons of the MNTB, which may suppress the effects of MOC activity on the cochlea.

## Introduction

Acoustic stimuli are processed through complex ascending and descending neuronal circuits for nuanced perception of sound. The final stage of descending auditory circuitry consists of medial and lateral olivocochlear (LOC) efferent neurons in the superior olivary complex (SOC; Rasmussen, 1946, 1960; Warr, 1975; Warr and Guinan, 1979; Guinan et al., 1983). The functions of medial olivocochlear (MOC) neurons are thought to inhibit cochlear activity via synapses onto cochlear outer hair cells (OHCs), and subsequently alter OHC electromotility and basilar membrane mechanics (Fex, 1967; Mountain, 1980; Siegel and Kim, 1982; Guinan, 1996, 2010; Elgoyhen and Katz, 2012). MOC neurons thereby exert a variety of downstream effects, including adjusting cochlear gain to respond to sound intensity spanning orders of magnitude (Galambos, 1956; Desmedt, 1962; Wiederhold and Peake, 1966; Wiederhold and Kiang, 1970; Geisler, 1974; Guinan and Gifford, 1988), improved hearing in background noise (Winslow and Sachs, 1987; Kawase et al., 1993), protection against noise-induced trauma (Rajan, 1988, 1995; Reiter and Liberman, 1995; Taranda et al., 2009; Maison et al., 2013; Tong et al., 2013; Boero et al., 2018), and auditory attention (Oatman, 1976; Glenn and Oatman, 1977; Delano et al., 2007; Terreros et al., 2016). Disorders such as tinnitus and hyperacusis may alter the function of MOC neurons (Attias et al., 1996; Knudson et al., 2014).

Synaptic activity driving and modulating MOC action potential patterns and subsequent auditory periphery effects are incompletely described because MOC somata are scattered among other SOC neurons, hindering localization for patch-clamp electrophysiology. *In vitro* experiments with *post hoc* identification from ventral nucleus of the trapezoid body (VNTB) neurons, which includes most MOC neurons, have elucidated some intrinsic electrical properties (Robertson, 1996; Fujino et al., 1997; Tong et al., 2013). The presynaptic neurons mediating sound-driven MOC activation have been inferred from sound responses; *in vivo* MOC axon recordings demonstrate sharp frequency tuning and “chopper” firing patterns (Fex, 1962a; Robertson, 1984; Robertson and Gummer, 1985; Liberman and Brown, 1986; Brown, 1989), that, with lesion and tracing studies, suggest primary sound-driven inputs from T-stellate cells of the ventral cochlear nucleus (VCN; Brown et al., 2003, 2013; de Venecia et al., 2005; Darrow et al., 2012). Anatomical and functional studies indicate additional synapses onto MOC neurons including from the inferior colliculus, auditory cortex, and nonauditory centers (Faye-Lund, 1986; Caicedo and Herbert, 1993; Thompson and Thompson, 1993; Vetter et al., 1993; Mulders and Robertson, 2000b, 2002; Groff and Liberman, 2003; Horváth et al., 2003; Ota et al., 2004; Gómez-Nieto et al., 2008; Brown et al., 2013; Suthakar and Ryugo, 2017). Histochemical staining for neuromodulators and morphological assessment of synapses suggest excitatory, inhibitory, and modulatory inputs to MOC neurons (Helfert et al., 1988; Thompson and Thompson, 1995; Woods and Azeredo, 1999; Mulders and Robertson, 2000a; Benson and Brown, 2006; Suthakar and Ryugo, 2017). Indeed, excitatory and inhibitory synaptic responses were reported in putative MOC neurons identified *post hoc* by dendrite morphology (Robertson, 1996). However, a more high-throughput method for locating MOC neurons is necessary to fully characterize the neuronal circuits that govern their activity to better understand the cochlear functions of MOC neurons under diverse acoustic conditions.

Here, we investigated the inhibitory synaptic inputs to MOC neurons. We validated a genetic labeling (ChAT-IRES-Cre mice crossed with a tdTomato reporter line) strategy to visualize MOC neurons and enable patch-clamp electrophysiology in brainstem slices. We demonstrated that MOC neurons receive GABAergic and glycinergic synaptic inputs. Further, we identified the medial nucleus of the trapezoid body (MNTB) as a source of inhibitory synapses, revealing a previously unknown MNTB–MOC connection. Trains of inhibitory synaptic inputs suppress action potentials and depress at stimulation rates relevant for sound, suggesting the release of MOC neurons from inhibition during sustained sound. The *in vivo* effect of MNTB inhibition of MOC neurons remains unexplored, but could have numerous roles, including suppression of the action potential rate of MOC neurons, providing a shunting hyperpolarization that sharpens the timing of responses of MOC neurons, or delaying MOC responses to prevent the suppression of cochlear responses to salient, rapidly changing stimuli.

## Materials and Methods

### 

#### 

##### Animals and slice preparation.

Animal procedures followed National Institutes of Health guidelines, as approved by the National Institute of Neurological Disorders and Stroke/National Institute on Deafness and Other Communication Disorders Animal Care and Use Committee. Brain slices were prepared from postnatal day 12 (P12) to P23 (majority of experiments) or P12—P36 (see Fig. 4*C*, experiments) mice of either sex resulting from a cross between ChAT-IRES-Cre transgenic mice on either a C57BL/6J (The Jackson Laboratory 028861) or a C57BL/6N (The Jackson Laboratory 018957) background strain, with tdTomato reporter mice (Ai14, Cre reporter allele inserted into Rosa 26 locus; catalog #007914, The Jackson Laboratory). Hemizygotes were used to prevent deleterious effects noted in ChAT-IRES-Cre homozygotes (Chen et al., 2018). As noted in the mouse line descriptions, these two ChAT-IRES-Cre strains are occasionally prone to ectopic expression of Cre due to the presence of the neo cassette. In some mice, this manifested as tdTomato expression in vasculature, glia, and other obvious cellular entities known to be noncholinergic (e.g., MNTB principal cell bodies and Calyces of Held). If any ectopic expression patterns were observed, the tissue from the animal was not used. Mice were killed by carbon dioxide inhalation at a rate of 20% of chamber volume per minute, then decapitated. The brain was removed in cold artificial CSF (aCSF) containing the following (in mm): 124 NaCl, 2 CaCl_2_, 1.3 MgSO_4_, 5 KCl, 26 NaHCO_3_, 1.25 KH_2_PO_4_, and 10 dextrose; 1 mm kynurenic acid was included during slice preparation. The pH was equal to 7.4 when bubbled with 95% O_2_/5% CO_2_. Where indicated, the aCSF calcium concentration was decreased to 1.2 mm. Three hundred micrometer transverse brain slices containing nuclei of the SOC including the MNTB and VNTB were cut with a vibratome (Leica) in cold aCSF. The slices were stored in a custom interface chamber at 32°C for 1 h and then allowed to cool to room temperature for electrophysiological recordings. Voltage-clamp recordings were performed at room temperature with the exception of glutamate uncaging experiments (see Fig. 5), which were performed at physiological temperature (35 ± 1°C). Current-clamp experiments were performed at physiological temperature. The slices were used within 4 h of preparation.

##### Retrograde label of efferent neurons.

**Figure 1.**
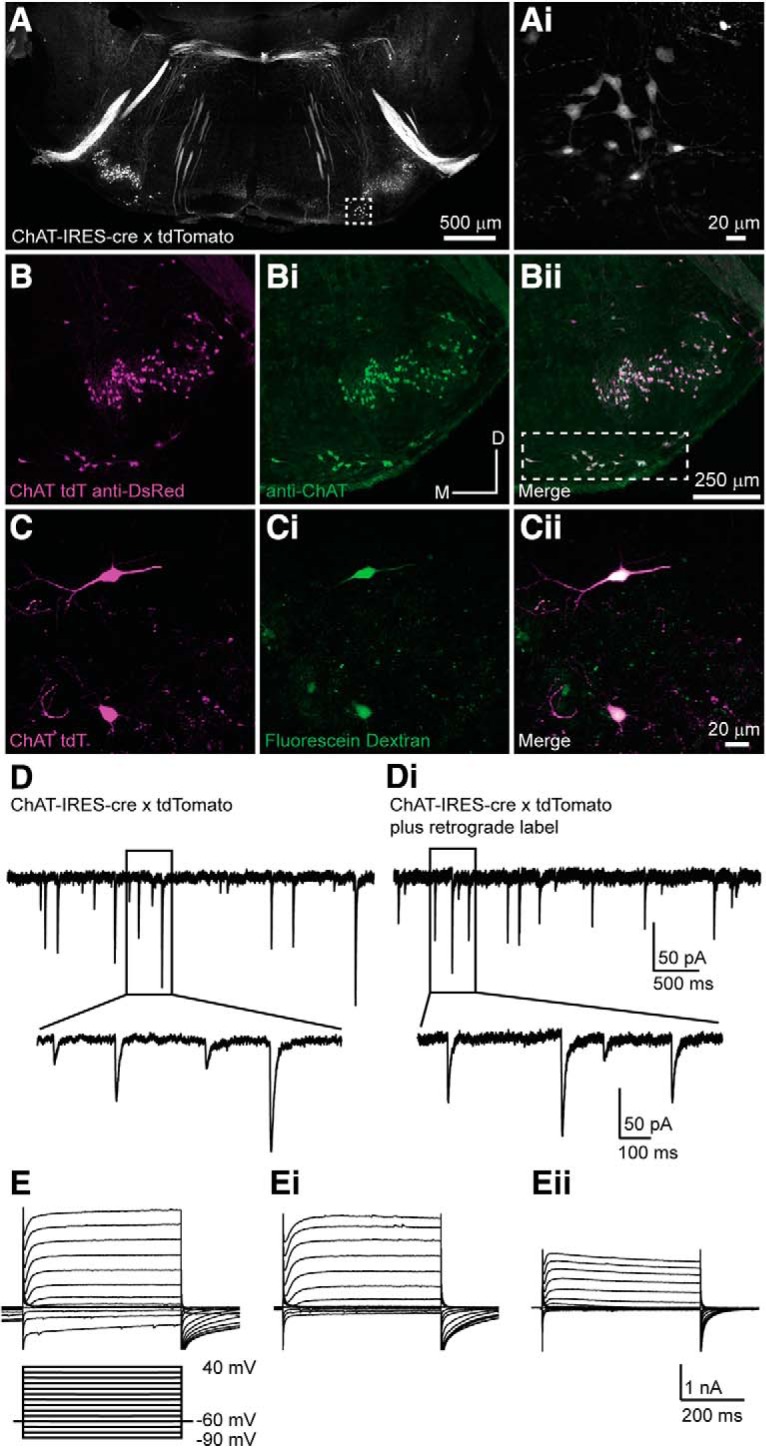
Validation of transgenic mouse line for MOC neuron localization *in vitro*. ***A***, Confocal image of a PFA-fixed transverse brain slice from a P17 ChAT-IRES-Cre × tdTomato mouse showing a pattern of fluorescent cells and fibers. Box indicates the approximate area of labeled MOC neurons in VNTB. ***Ai***, Zoom image of the area indicated in ***A***. ***B***, Anti-DsRed antibody (magenta) fluorescence of neurons in the SOC from a P30 ChAT-IRES-Cre × tdTomato mouse. ***Bi***, Anti-ChAT antibody (green) immunofluorescence. ***Bii***, Merged image of ***B*** and ***Bi*** showing colocalization (white) of ChAT-IRES-Cre tdTomato fluorescence (amplified with anti-DsRed antibody) and anti-ChAT antibody labeling. Region of the dashed box indicates MOC neurons. ***C***, Native fluorescence of two VNTB neurons from a P17 ChAT-IRES-Cre × tdTomato mouse (false color magenta). ***Ci***, Neurons labeled by acute application of green fluorescent dextran fluorescein to the cochlea followed by retrograde diffusion to the soma. ***Cii***, Merged image of ***C*** and ***Ci*** showing colocalization of genetic (magenta) and retrograde tracer (green) labels. ***D***, Voltage-clamp trace (*V*_m_ = −60 mV) from a red-labeled neuron in a ChAT-IRES-Cre × tdTomato mouse brainstem slice. Zoom image shows waveform of sPSCs. ***Di***, Voltage-clamp trace (*V*_m_ = −60 mV) from a neuron in the VNTB with both a genetic tracer (ChAT-IRES-Cre × tdTomato) and a retrograde tracer (dextran fluorescein) label. Zoom image shows waveform of sPSCs, which are similar to those in ***D***. ***E–Eii***, Example voltage-clamp traces from representative cells, QX-314 present in internal solution blocked voltage-gated sodium channels. The voltage-step protocol detailed under ***E*** applies to all panels.

In a subset of experiments, MOC somata were labeled via acute application of a fluorescent retrograde neuronal tracer (dextran fluorescein, 3000 molecular weight; Thermo Fisher Scientific) to the cochlea. The mice were killed as described above, decapitated, then partially dissected, exposing most of the brain but leaving the temporal bones and a portion of skull attached. The bulla was removed, and the cochlear modiolus was exposed. Dextran crystals were applied to the modiolus with a glass pipette to stain cochlear tissue and neuronal projections to the cochlea, including efferent axons. The brain with attached cochlea was supported sideways in bubbling aCSF so that brain tissue was submerged, and the cochlea barely protruded from the aCSF to keep tracer crystals within the cochlea. The brain was incubated at room temperature for 30 min to allow retrograde tracer transport, followed by the removal of remaining bone and normal slice preparation as described above for patch-clamp electrophysiology experiments. For high-resolution images of the colocalization of fluorescein-dextran crystal labeling with ChAT-IRES-Cre × tdTomato labeling, confocal images of live, unfixed MOC neurons were obtained on a Nikon A1R microscope (NIR Apo 60×/1.0 W) using NIS-Elements AR version 4.40.00 imaging software (Nikon). Images are maximum intensity projections from *z*-stacks (Fig. 1*C*).

##### Patch-clamp electrophysiological recordings.

Brain slices were transferred to a recording chamber continuously perfused at a rate of ∼2–3 ml/min with aCSF bubbled with 95% O_2_/5% CO_2_. The slices were viewed using a Nikon FN-1 Microscope with differential interference contrast (DIC) optics and a Nikon NIR Apo 40×/ 0.80 numerical aperture (NA) water-immersion objective. The images were collected with a QIClick, Mono 12 bit, noncooled camera (Nikon) and viewed using NIS-Elements software (Nikon). MOC neurons were identified for whole-cell voltage-clamp or current-clamp recordings by their position in the VNTB (MOC neurons in the dorsal periolivary (DPO) region were not recorded from in this study) and visibility using red epifluorescence (546 nm emission filter; SOLA light engine, Lumencor). The recordings were performed using a MultiClamp 700B amplifier and a DigiData 1440A digitizer controlled by Clampex 10.6 software (Molecular Devices) or a HEKA EPC10 amplifier controlled using PatchMaster version 2x90.4 or PatchMaster NEXT version 1.1. The recordings were sampled at 50 kHz and filtered on-line at 10 kHz. The internal solution for MOC voltage-clamp recordings contained the following (in mm): 56 CsCl, 44 CsOH, 49 d-gluconic acid, 1 MgCl_2_, 0.1 CaCl_2_, 10 HEPES, 1 EGTA, 0.3-Na-GTP, 2 Mg-ATP, 3 Na_2_-phosphocreatine, 5 QX-314, and 0.25% biocytin. Fisher Scientific Alexa Fluor-488 hydrazide or Alexa Fluor-350 hydrazide (10 μm; Thermo Fisher Scientific) was included. pH was adjusted to 7.2 with CsOH. The Cl^−^ equilibrium potential was approximately −20 mV. The internal solution for MOC neuron current-clamp recordings contained the following (in mm): 125 K-gluconate, 5 KCl, 1 MgCl_2,_ 0.1 CaCl_2,_ 10 HEPES, 1 EGTA, 0.3 Na-GTP, 2 Mg-ATP, 1 Na_2_-phosphocreatine, 0.25% biocytin, and 0.01 Alexa Fluor-488 hydrazide (Thermo Fisher Scientific). The pH was adjusted to 7.2 with KOH. The Cl^−^ equilibrium potential was −74 mV. The internal solution for glutamate uncaging contained the following (in mm): 76 Cs-methanesulfonate, 56 CsCl, 1 MgCl_2_, 1 CaCl_2_, 10 HEPES, 10 EGTA, 0.3 Na-GTP, 2 Mg-ATP, 5 Na_2_-phosphocreatine, 5 QX-314, and 0.01 Alexa Fluor-488 hydrazide; 0.25% biocytin was added in some experiments. The pH was adjusted to 7.2 with CsOH.

Recording pipettes were pulled from 1.5 mm outer diameter borosilicate glass (Sutter Instrument) to tip resistances of 3–6 MΩ. Series resistances were corrected 50–85%. The cells were voltage clamped at −60 mV, unless stated otherwise. Membrane voltages were not adjusted for a measured liquid junction potential of −2 mV (Cs gluconate solution) or −6 mV (Cs methanesulfonate solution). MNTB axons were electrically stimulated to evoke neurotransmitter release and to generate postsynaptic currents (PSCs) in MOC neurons by a large-diameter glass pipette of ∼10 or ∼30 μm diameter filled with aCSF connected to an Iso-Flex Stimulus Isolation Unit (A.M.P.I.), placed in the MNTB axon bundle within or at the lateral edge of the MNTB. MNTB axons were stimulated at 0.2 Hz with a current amplitude of 6–8000 μA. In the vast majority of experiments (all experiments using electrical stimulation of presynaptic axons except for Fig. 4*C*), the stimulus intensity was turned down to an intermediate intensity (280 ± 210 μA). Drugs were bath applied by addition to the recirculating aCSF solution. The drugs were obtained from Thermo Fisher Scientific or Millipore Sigma.

##### Glutamate uncaging.

Glutamate uncaging experiments were performed on a Nikon Eclipse Ni-E microscope with an Apo LWD 25×/1.10 NA water-immersion objective. DIC and epifluorescence images were captured using a Retiga Electro CCD camera (QImaging) on NIS Elements software (version 4.51.01). MNI-caged-L-glutamate (0.2 mm in aCSF; Tocris Bioscience) was bath applied. The bath temperature was held at 35 ± 1°C using an in-line heater (Warner) coupled to a temperature controller (Warner). A 100 μm 0.37 NA optical fiber (Prizmatix) was positioned over the MNTB and connected to a 365 nm LED light source (Prizmatix). The approximate power at the surface of the slice was measured at ∼24 mW/mm^2^. After collecting baseline data for spontaneous PSCs (sPSCs), glutamate was uncaged using three 50 ms LED pulses (10 ms interpulse interval). The fiber was systematically moved around the slice surface above the MNTB, and the uncaging was repeated (see Fig. 5*B*, approximate grid). In each experiment, the slice was positioned in the bath so that the optical fiber was perpendicular to the long (mediolateral) axis of the MNTB. With this orientation, the fiber was driven along this axis in ∼100 μm increments (width of the optical fiber). Generally, the fiber was initially placed at one “corner” of the MNTB and then moved mediolaterally to the opposite side along the edge closest to the fiber (usually, five discrete locations) such that the illumination was focused within one-half of the MNTB in the dorsoventral axis. Then, the fiber was moved to the middle of the MNTB in the dorsoventral dimension, and the fiber was progressed across the nucleus again (for a total of 10 locations). Typically, the fiber was then positioned near the dendrites of the MOC neuron to induce a direct glutamate response as a comparison to the synaptically evoked responses from MNTB stimulation. Uncaging was performed at −60 mV and, in separate experiments, at 0 mV, the approximate reversal potential for AMPA-mediated glutamatergic currents.

##### Biocytin histology.

Neurons that were filled with biocytin during recordings were subsequently processed for DAB to visualize neuronal morphology. After patch-clamp recordings were completed, slices were fixed by immersion in 4% paraformaldehyde (PFA; Electron Microscopy Sciences) in PBS buffer for 1–5 d at 4°C, then rinsed and stored in 1× PBS followed by overnight cryoprotection in 30% sucrose in PBS. The slices were frozen and thawed three times on dry ice, followed by DAB staining according to the manufacturer instructions (Vectastain ABC Kit, Vector Laboratories). The sections were dried on gelatin-coated slides, dehydrated in ethanol, cleared in xylenes, then coverslipped in Permount Mounting Medium (Thermo Fisher Scientific) for imaging.

##### Immunohistochemistry.

A P26 and a P30 ChAT-IRES-Cre × tdTomato mouse were anesthetized via intraperitoneal injection of ketamine (120 mg/kg) and xylazine (25 mg/kg). The animals were then transcardially perfused with 1× PBS, followed by 4% PFA in PBS buffer. After perfusion, the brains were removed and postfixed overnight at 4°C in 4% PFA. Coronal sections (40 μm) were cut using a Microm freezing microtome (Thermo Fisher Scientific). Floating sections were incubated in blocking solution (1% bovine serum albumin, 0.5% Triton X-100 in PBS) for ∼30 min at room temperature. Primary antibodies [1:500; goat anti-choline acetyltransferase (ChAT), catalog #AB144P, Millipore; 1:1000; rabbit anti-DsRed, catalog #632496, Takara] were prepared in blocking solution. The sections were incubated with primary antibodies for ∼48 h at 4°C. The sections were then rinsed in PBS before a 24 h incubation with secondary antibodies at 4°C (1:500; Alexa Fluor 488 donkey anti-goat IgG, catalog #711–165-152, Jackson ImmunoResearch; 1:500; Cy3 donkey anti-rabbit IgG, catalog #705–545-003, Jackson ImmunoResearch). After secondary incubation, sections were rinsed and mounted on slides (Fluoromount G, SouthernBiotech). Confocal images were acquired on a Nikon Eclipse Ti–A1R inverted microscope using NIS Elements software (version 4.51.01). Maximum intensity projections were created from *z*-stacks. To quantify the colocalization of the genetically labeled ChAT-expressing neurons (ChAT-IRES-cre × tdTomato-positive cells, amplified with anti-DsRed) with anti-ChAT antibody, two blinded observers manually counted labeled cells in 10 slices from two mice. The bilateral images contained the VNTB and were cropped to exclude the lateral superior olive (LSO), where LOC cells reside. Cell counts were performed on monochrome grayscale images of both the anti-DsRed and the anti-ChAT antibody staining, as well as merged images. The anti-DsRed and anti-ChAT antibodies clearly colocalized in 92% of the cells in the merged images. The occurrence of singly labeled neurons was low for both anti-DsRed (3.6%) and anti-ChAT antibody (4.3%). The rarity of anti-DsRed cells that did not appear to be cholinergic with antibody staining (i.e., red only) suggests either that some MOC cells have faint anti-ChAT antibody staining or that occasionally noncholinergic, and potentially non-MOC cells, are tdTomato positive in this mouse line. Cell counts in the monochrome channels were consistent with the merged numbers (merges, 139.5 cells; anti-DsRed, 143.5 cells; anti-ChAT antibody, 128 cells).

##### Experimental design and statistical analysis.

For a comparison of properties of genetically labeled (ChAT-IRES-Cre × tdTomato) cells to those that were labeled by both genetic cross and retrograde fluorescein dextran label, we randomly selected 10 age- and sex-matched pairs of animals, with one neuron recorded per animal. Six males and four females were selected per group. Cellular characteristics (e.g., membrane capacitance, input resistance, sPSC rate) were compared between the two groups, which are presented in Table 1.

**Table 1.** Comparison of characteristics of VNTB neurons identified by genetic label (ChAT-IRES-Cre × tdTomato) or with genetic label and retrograde tracer label (dextran fluorescein) from the cochlea

	ChAT-IRES-Cre × dT	Retrograde tracer + ChAT-IRES-Cre × dT	*p* Value
Sex of mice for selected neurons	6 male, 4 female	6 male, 4 female	N/A
Postnatal age (d)	15.5 ± 3.5	16 ± 3	1
Input resistance (MΩ)	183.3 ± 92.9	170.0 ± 88.0	0.97
Membrane capacitance (pF)	25.5 ± 5.7	33.8 ± 3.6	0.11
sPSC rate (/s)	0.62 ± 0.42	1.03 ± 0.70	0.36
sPSC amplitude (pA)	41.4 ± 4.6	51.6 ± 10.9	0.21
Proportion receiving evoked PSCs	8 of 10	7 of 10	N/A
Convergence ratio	5.2 ± 1.8	4.0 ± 1.7	0.82

Ten retrograde-labeled cells were age and sex matched with a random selection of 10 genetically labeled neurons. Data are the median ± median absolute deviation.

Spontaneous PSCs were automatically identified using MiniAnalysis software version 6.0.7 (Synaptosoft), using a threshold of 2× RMS noise. PSCs were then accepted or rejected based on the characteristic PSC waveform. The decay time constants of PSCs were calculated in Mini Analysis software from individual events. Evoked PSC amplitudes were measured in Clampfit version 10.6 (Molecular Devices) by manually placing cursors at the immediate onset of the PSC (either at the baseline or, during trains of stimuli, during the decay of the preceding PSC), and at the peak of the PSC. The change in kinetics of PSCs in drug-treated conditions was compared using the final 3 min (of 7–10 min) of each condition to ensure that all drugs had time to take effect.

To determine the number of MNTB axons synapsing onto an MOC neuron, we measured the convergence ratio (CR) by dividing the maximum evoked PSC amplitude by the minimum evoked PSC amplitude (Kim and Kandler, 2003). However, because this method is insensitive to different presynaptic axons evoking postsynaptic responses of varying magnitudes, we also performed a k-means clustering analysis of evoked PSC amplitudes in the experiments in which we performed fine-grained increases in electrical stimulus intensity (Ferragamo et al., 1998). The mean distance of the amplitude of each evoked PSC (ePSC) from the cluster center was plotted by the number of clusters, then the elbow method was used to determine the appropriate number of clusters (i.e., number of MNTB axons).

To test connectivity between MNTB and MOC neurons using glutamate uncaging, control data were collected by recording voltage-clamp sweeps in MOC neurons with no uncaging stimulus. PSCs were detected using Mini Analysis software (detection threshold, 2× rms noise). For the glutamate-uncaging stimulation condition, PSCs were counted during a time window that started with the onset of the first light pulse and ended after 200 ms. For the 0 mV holding potential experiments, the light pulse was delayed until the response to the 0 mV current step reached steady state. In these experiments, control data were collected during the 400 ms time window from 3.6 to 4.0 s after the initial voltage step to 0 mV. To determine the approximate percentage of MOC neurons receiving MNTB input evoked by glutamate uncaging, we arbitrarily defined an evoked input as an experiment in which light stimulus evoked an increase in PSC rate of >50%. This figure was then used to compare with the proportion of cells receiving MNTB inputs evoked by electrical stimulation.

In experiments to measure the effect of MNTB synaptic inhibition on MOC action potentials, first the IPSC was recorded in voltage-clamp, and an electrical stimulation amplitude was selected that evoked reliable, but intermediate-amplitude, responses (as described above). Cells were omitted if the electrical stimulation also directly evoked action currents or action potentials in the MOC neuron, presumably due to retrograde stimulation of the MOC axon. The recording configuration was then switched to current-clamp, and the MNTB axon stimulation was repeated in single pulses to measure the IPSP. Action potentials recorded in current-clamp were measured in Clampfit version 10.6 using the Threshold Search function to determine the peak time of each action potential. The latency to action potential was measured in the axon stimulation window beginning at the first electrical pulse, and within the control window, beginning exactly 5 s after the first electrical pulse.

All statistical analyses were performed using Origin version 2019 (Origin Laboratory). Data were examined for normal distribution using the Shapiro–Wilk test. Nonparametric tests were applied given the non-normal distribution of many variables studied. The difference between a population median and a set value (0 or 1 for normalized or ratio measurements) was tested with a one-sample Wilcoxon signed-rank test. The differences between two paired groups/conditions were assessed using a Wilcoxon signed-rank test. The differences between two independent groups were assessed using a Mann–Whitney *U* test. The differences among multiple groups were evaluated using the Kruskal–Wallis ANOVA test, followed by Mann–Whitney *U* test with Bonferroni correction for multiple comparisons. The change in PSC decay time constant, or MNTB–MOC convergence ratio, by postnatal age was tested using a linear regression fit to the data. A relationship between the data and postnatal age was considered significant if the linear fit had a *p* value < 0.05. The differences were considered significant if *p* < 0.05. For data presentation, voltage-clamp traces of single sweeps were filtered to 2 kHz for Figures 1, 3, 4, and 7. The traces were not filtered in Figure 5 or 8. In cases where more than one trace was averaged, the data were not filtered. Summary box plots indicate the median and quartiles, with 10th and 90th percentiles indicated by error bars. A square within the box plot indicates the mean. Individual data points are overlaid. Most data are presented as the median ± median absolute deviation. Some data were normally distributed and therefore presented as the mean ± SD, as indicated in the text. The figures were prepared in Origin version 2019 and Adobe Illustrator CC 2018 (Adobe Systems). Asterisks in figures indicate *p* < 0.05, exact values are given in the results text.

## Results

### Localization of MOC neurons for patch-clamp recordings

MOC efferent neuron somata are diffusely located in the VNTB and DPO regions of the SOC (Warr, 1975; Warr and Guinan, 1979; Guinan et al., 1983) and are difficult to identify in unstained preparations among other neurons. MOC neurons are cholinergic; therefore, we used ChAT-IRES-Cre mice in which cholinergic neurons express Cre recombinase crossed with the Ai14 tdTomato reporter mouse line that expresses the gene for tdTomato in Cre-expressing cells (Fig. 1*A–C*). This enabled us to localize MOC neurons in brain slices for patch-clamp electrophysiology experiments. To validate the expression pattern of this genetic model, PFA-fixed coronal sections containing the SOC were prepared from P26 and P30 ChAT-IRES-Cre × tdTomato mice and stained with an antibody against ChAT (Fig. 1*B*). tdTomato fluorescence (amplified with anti-DsRed antibody; Fig. 1*B*, magenta) colocalized with anti-ChAT antibody labeling in the VNTB (Fig. 1*Bii*, boxed region), where the majority of MOC neurons are found, confirming the expected expression patterns of the Cre recombinase.

For patch-clamp electrophysiological recordings, transverse brainstem slices including the regions of the SOC were prepared from ChAT-IRES-Cre × tdTomato mice aged P12–P23, and red fluorescent cells in the VNTB were identified using epifluorescence illumination. To further validate the genetic model and to confirm the identity of red fluorescent neurons in the VNTB, a subset of recordings was performed in slices from animals in which a green dye (dextran fluorescein) was acutely applied to MOC axons in the cochlea with retrograde transport to the soma, followed by brain dissection and typical slicing procedures (see Materials and Methods). With this acute procedure, few neurons were usually labeled and were often dim, but green-labeled cells in the VNTB were also red (Fig. 1*C–Cii*, magenta), confirming axon projections of tdTomato-expressing cells to the cochlea. Intrinsic electrical properties of MOC neurons, including cell capacitance and input resistance, were compared in age- and sex-matched neurons targeted with either the genetic label alone or combined genetic and retrograde labeling (Fig. 1*C–Cii*). Cells with combined labeling were indistinguishable from cells with only genetic labeling (Fig. 1*D*,*Di*, Table 1), further indicating that genetic and retrograde labels target the same population of cells, and that genetically labeled cells in the VNTB from ChAT-IRES-Cre × tdTomato mice are MOC neurons.

**Figure 2.**
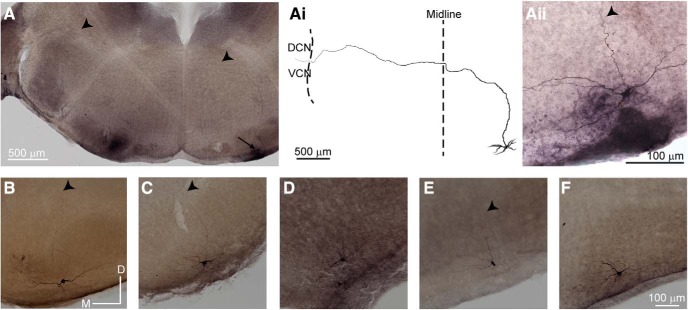
Morphology of labeled neurons in the VNTB from ChAT-IRES-Cre × tdTomato mice is consistent with MOC neurons. ***A–F***, Three hundred micrometer transverse brain slices from a ChAT-IRES-Cre × tdTomato mouse, from which a VNTB neuron patch-clamp recording was performed. Neuron morphology visualized from biocytin diffusion into the cell during patch-clamp recording followed by PFA fixation and DAB processing. ***A***, Tiled image of a slice, with MOC somata indicated (arrow) and a filled axon visible (arrowheads) projecting to contralateral cochlear nucleus. ***Ai***, Tracing of axon from ***A*** showing characteristic MOC morphology for an “ipsilateral” neuron. DCN, Dorsal cochlear nucleus. ***Aii***, Zoom image of soma and dendrites for the neuron in ***A***. ***B–F***, Example MOC neurons showing soma and dendrite morphology, with axons indicated if visible (arrowheads).

To further validate the ChAT-IRES-Cre mouse line, we compared the morphology of neurons from which we performed recordings to examples of MOC neurons from the literature. MOC neurons were filled with biocytin (0.25%) during recordings, after which the tissue was fixed in PFA and stained with DAB (see Materials and Methods). A majority of neurons (96 of 151) had large (round/pyramidal) somata with either multipolar dendrites (66 neurons) or dendrites projecting mediolaterally (30 neurons; Fig. 2*Aii*,*C*,*D*,*F*), consistent with previous reports (Vetter and Mugnaini, 1992; Yao and Godfrey, 1998; Brown and Levine, 2008). The remainder of neurons (55 of 151) had elongated cell bodies, also with mediolateral dendritic projections (Fig. 2*B*,*E*). In some neurons (41 of 151), a portion of the axon was visible and projected dorsally toward the fourth ventricle, as previously described, but usually the tracer faded or the axon was cut at the slice surface with a terminal axonal bleb (Fig. 2*B*, *C*, *E*). However, in two cells the axon fill could be traced to the contralateral cochlear nucleus, following characteristic axon projection patterns of ipsilateral MOC neurons (double crossed, they receive most sound information from the same cochlea that the axon projects to; Fig. 2*A*). MOC neurons are known to innervate cochlear and vestibular nuclei (Rasmussen, 1960; Brown et al., 1988, 1991; Winter et al., 1989; Ryan et al., 1990; Brown, 1993; Benson and Brown, 1996; Benson et al., 1996; Horváth et al., 2000; Baashar et al., 2019), but the axon fill faded before cochlear nucleus targets could be approximated. Collectively, antibody labeling, retrograde tracing, and neuronal morphology experiments confirm that the ChAT-IRES-Cre mouse line represents a viable way to identify and perform patch-clamp recordings from MOC neurons.

### Synaptic inputs to MOC neurons

**Figure 3.**
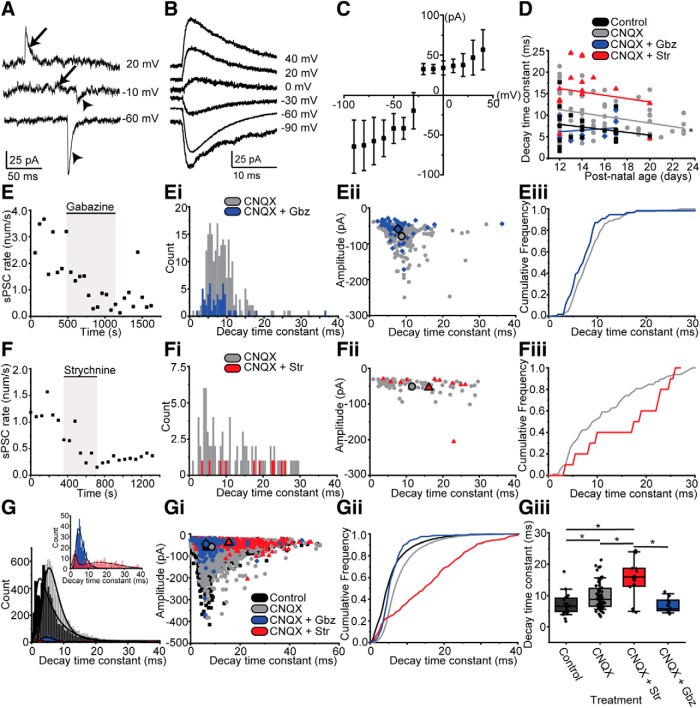
MOC neurons receive glutamatergic, GABAergic, and glycinergic innervation. ***A***, sPSCs recorded in MOC neurons at the holding potentials indicated. Arrowheads, inward sPSCs; arrows, outward sPSCs. ***B***, Average waveforms of sPSCs recorded from an example MOC neuron at the holding potentials indicated (in 5 μm CNQX). Each waveform is the average of 6 (0 mV) to 89 (−60 mV) sPSCs. ***C***, Current–voltage relation of the average amplitude of sPSCs at different holding potentials as in ***B***; (in 5 μm CNQX) from 12 neurons. sPSC reversal potential is approximately −20 mV. ***D***, Scatterplot of sPSC time constant of decay by postnatal age in different postsynaptic receptor blockers. ***E–Eiii***, Analysis of sPSC rate and kinetics for a single representative MOC neuron treated with gabazine. ***E***, sPSC rate plotted by time in CNQX (5 μm), gabazine (30 μm, shaded box), and wash. Each data point represents the sPSC rate for 1 min. ***Ei***, Frequency histogram of sPSC time constant of decay in CNQX (gray) and with the addition of gabazine (blue). ***Eii***, Scatterplot of sPSC amplitude by time constant of decay in control (CNQX, gray circles) and the addition of gabazine (blue diamonds). Large points outlined in black indicate average. ***Eiii***, Cumulative frequency histogram of the time constant of decay for sPSCs in CNQX (gray) and with the addition of gabazine (blue). ***F–Fiii***, Analysis of sPSC rate and kinetics for a single representative MOC neuron treated with strychnine (red), using the same format as in ***E–Eiii***. ***G–Giii***, Summary data for sPSC pharmacology. ***G***, Frequency histogram of the time constant of decay for all neurons, pooled. Black lines are LogNormal fits to data, except for CNQX plus strychnine, which is fit with two Gaussian distributions. Inset is a zoom of CNQX plus gabazine and CNQX plus strychnine data. ***Gi***, Scatterplot of sPSC amplitude plotted by the time constant of decay, with average values per drug treatment indicated by large points outlined in black. ***Gii***, Cumulative frequency histogram of all cells, pooled. ***Giii***, Summary data of decay kinetics, with all cells pooled. In all panels an asterisk indicates *p* < 0.05.

Patch-clamp recordings from MOC neurons in ChAT-IRES-Cre × tdTomato mouse slices were performed in voltage-clamp to assess synaptic inputs to the neurons (Fig. 1*E–Eii*). At a holding potential of −60 mV, sPSCs were recorded (Figs. 1*D*, 3*A*). The membrane potential was stepped to different voltages between −90 and +40 mV, in 10 mV increments. At membrane voltages from −90 to −30 mV, PSCs were entirely inward. From 0 to +40 mV, PSCs were entirely outward. However, at intermediate voltages of approximately −10 mV (liquid junction potential not corrected), both inward and outward sPSCs were recorded (*n* = 8 neurons; Fig. 3*A*). This suggests that at these intermediate membrane voltages, sPSCs with different polarities are due to the activation of different postsynaptic receptors with different ionic permeabilities, most likely a mix of EPSCs and IPSCs.

Postsynaptic receptors were then pharmacologically blocked to identify the neurotransmitters generating EPSCs and IPSCs in MOC neurons. With the high chloride concentration used in the internal solution, both EPSCs and IPSCs were inward and of similar amplitude. Therefore, the rate of sPSCs was quantified to detect a drug effect instead of PSC amplitude. Bath application of the ionotropic glutamate receptor blocker 6-cyano-7-nitroquinoxaline-2,3-dione (CNQX; 5 μm) significantly reduced the sPSC rate (control aCSF: 2.68 ± 1.6 /s; CNQX: 0.82 ± 0.46/s; one-tailed Wilcoxon signed-rank test: W = 219, *n* = 21 neurons, *p* = 3.3E-5), confirming glutamatergic synapses onto MOC neurons mediated by postsynaptic AMPA receptors. Voltage steps from −90 to +40 mV were again applied. The reversal potential of remaining sPSCs was approximately −20 mV (Fig. 3*B*,*C*), the approximate reversal potential for chloride ions, suggesting inhibitory neurotransmission. Next, we tested the neurotransmitters mediating inhibitory neurotransmission onto MOC neurons. First, AMPA receptors were blocked with CNQX. The sPSC rate was reduced by gabazine (SR 95531), a GABA_A_ receptor blocker (CNQX: 1.91 ± 1.06/s; 30 μm gabazine: 0.24 ± 0.11/s; one-tailed Wilcoxon signed-rank test: W = 55, *n* = 10 neurons, *p* = 9.8E-4; wash to 0.49 ± 0.18/s after 7 min, one-tailed Wilcoxon ranked sum test, W = 1, *n* = 7 neurons, *p* = 0.0156; Fig. 3*E*). In another set of experiments, the sPSC rate was reduced by bath application of the glycine receptor antagonist strychnine (CNQX: 0.76 ± 0.56/s; 1 μm strychnine: 0.25 ± 0.22/s; one-tailed Wilcoxon signed-rank test: W = 103, *n* = 22 neurons, *p* = 0.0062; wash to 0.32 ± 0.25/s after 7 min; one-tailed Wilcoxon signed-rank test: W = 0, *n* = 9 neurons, *p* = 0.00391; Fig. 3*F*). Sequential application of CNQX, gabazine, and strychnine nearly eliminated sPSCs (0.03 ± 0.03/s, *n* = 9 neurons). In sum, MOC neurons receive both excitatory and inhibitory synaptic inputs. Excitatory inputs are glutamatergic, mediated by postsynaptic AMPA receptors. Inhibitory inputs are either glycinergic or GABAergic and mediated by ionotropic glycine or GABA_A_ receptors.

We quantified the kinetics of sPSCs recorded in the presence of different receptor blockers from the above experiments to determine the relative time course of different postsynaptic receptor currents. We compared the time constant of decay (τ), pooled across all experiments, for control PSCs (control τ: 6.56 ± 2.11 ms; skew = 2.32; LogNormal fit, *r*^2^ = 0.93, *p* ≪ 0.0001; center: 5.24 ms; Log SD: 0.83 ms; *n* = 21 neurons; 195 ± 132 PSCs/neuron), mixed inhibitory receptor PSCs (CNQX: 8.74 ± 2.47 ms; skew = 2.17; LogNormal fit *r*^2^ = 0.98; *p* ≪ 0.0001; center: 6.59 ms; Log SD: 0.43 ms; *n* = 72 neurons; 55 ± 42.5 PSCs/neuron), GABAergic receptor PSCs (CNQX and strychnine: 15.90 ± 2.78 ms; skew = 0.52; double Gaussian fit: *r*^2^ = 0.71, p ≪ 0.0001; peak 1: center: 2.56 ms; width: 2.11 ms; peak 2: center: 16.36 ms; width: 20.92 ms; *n* = 16 neurons; 19 ± 12.5 PSCs/neuron) or glycinergic receptor PSCs (CNQX and gabazine: 5.76 ± 1.33 ms; skew = 4.22; LogNormal fit *r*^2^ = 0.91; *p* ≪ 0.0001; center: 5.48 ms; Log SD: 0.41 ms; *n* = 10 neurons; 27 ± 17 PSCs/neuron). Mixed inhibitory PSC kinetics (Fig. 3*Giii*, gray) were significantly longer than control PSC kinetics (Fig. 3*Giii*, black), suggesting that inhibitory synaptic responses are slower than excitatory synaptic responses. The magnitude of the difference is assumed to be an underestimate because the control condition contains both excitatory and inhibitory synaptic responses. Isolated GABAergic PSCs (Fig. 3*Giii*, red) were significantly slower than isolated glycinergic PSCs [Fig. 3*Giii*, blue; Kruskal–Wallis ANOVA, χ^2^ = 22.82318 (3 df), *p* = 4.4E-5; *post hoc* Mann–Whitney pairwise comparisons with Bonferonni correction, significance cutoff, *p* < 0.0083; Fig. 3*Giii*]. MOC recordings were performed at ages during which the morphological and postsynaptic receptor properties of mouse auditory neurons in the SOC are still undergoing the final steps of maturation (Kandler and Gillespie, 2005; Kandler et al., 2009). To determine whether postsynaptic receptors in MOC neurons are undergoing developmental changes, we plotted the sPSC decay time constant by animal age. We observed a slight but significant decrease in the kinetics of mixed inhibitory PSCs (CNQX; Fig. 3*D*; *r*^2^ = 0.082, *p* = 0.0086). However, there were no significant changes in the kinetics of the isolated GABAergic or glycinergic PSCs with development in this age range.

**Figure 4.**
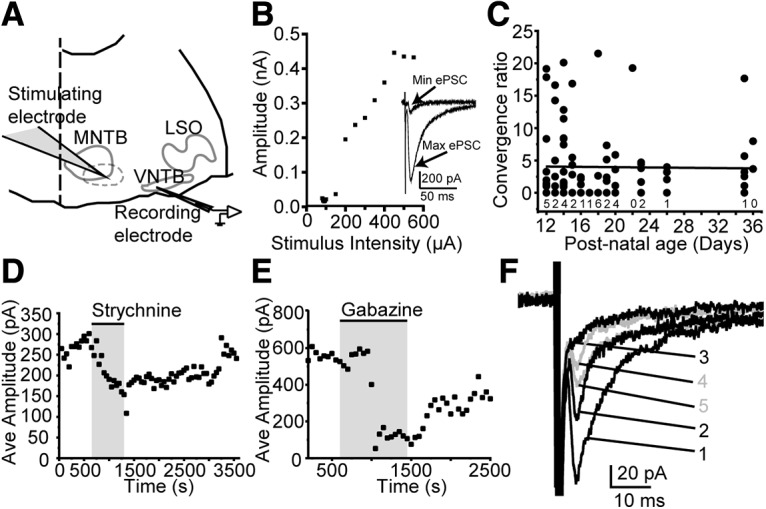
PSCs evoked from axons proximal to the MNTB are glycinergic and GABAergic. ***A***, Schematic of locations for patch-clamp recording and electrical axon stimulation. Stimulating electrode placed in area indicated by dashed line. ***B***, Plot of ePSC amplitude by stimulus intensity for electrical axon stimulation, single neuron. Inset, Example maximum and minimum ePSCs from a representative neuron. ***C***, Plot of convergence ratio by age. Numbers under the black circles indicate the number of neurons with no evoked PSCs per age. ***D***, ***E***, Plot of average ePSC amplitude by time; each data point is the average of 10 PSCs. CNQX (5 μm) is present in the bath solution. Drugs [strychnine (1 μm; ***D***) and gabazine (30 μm; ***E***)] added to bath solution during the period indicated by the shaded box. ***F***, Example PSCs evoked from electrical stimulation of presynaptic axons in a representative MOC neuron. CNQX (5 μm) is present in the bath solution. Numbers indicate the order in which traces were recorded: 1, control (with CNQX); 2, addition of strychnine; 3, strychnine plus gabazine; 4, strychnine remains, wash of gabazine; 5, wash of strychnine, CNQX remains in the bath solution. Strychnine application reduced the ePSC amplitude. The subsequent addition of gabazine nearly eliminated the ePSC. Gray traces indicate recovery of the ePSC waveform after a wash of gabazine and strychnine.

Our electrophysiology results are in line with previous anatomical studies suggesting that, in addition to excitatory synapses (Helfert et al., 1988; Benson and Brown, 2006; Suthakar and Ryugo, 2017), modulatory or inhibitory synapses onto MOC neurons are also present (Helfert et al., 1988; Thompson and Thompson, 1995; Woods and Azeredo, 1999; Mulders and Robertson, 2000a; Benson and Brown, 2006). Further, a report of IPSPs in putative MOC neurons identified *post hoc* by axon and dendrite morphology (Robertson, 1996) is also corroborated by our data. However, the source of potential synaptic inhibition of MOC neurons is unknown. To identify the presynaptic neurons giving rise to inhibitory synapses, we again recorded from MOC neurons and performed electrical stimulation of presynaptic axons via a wide-bore pipette delivering current generated by a stimulus isolation unit to drive neurotransmitter release. CNQX (5 μm) was included in the bath to block AMPA receptors. Electrical stimulation within or lateral to the MNTB (Fig. 4*A*, dashed line, schematic), a location targeted for activating MNTB axons projecting to the LSO (Kotak et al., 1998; Kim and Kandler, 2003, 2010; Weisz et al., 2016), reliably evoked PSCs in 69 of 117 MOC neurons, suggesting functional connectivity between the MNTB and the MOC. Indeed, the MNTB is a likely source of inhibitory synapses to the MOC, as it provides innervation to many other auditory nuclei (Morest, 1968; Kuwabara and Zook, 1991, 1992; Sanes and Siverls, 1991; Smith et al., 1998) and is inhibitory (Moore and Caspary, 1983; Adams and Mugnaini, 1990; Bledsoe et al., 1990; Wu and Kelly, 1991). Stimulation was performed at 0.2 Hz at a range of stimulation intensities. Minimal stimulation (≥50% evoked PSC failures; stimulation range, 6–500 μA) was used to activate single presynaptic axons, yielding ePSCs (27.34 ± 9.14 pA; *n* = 60 neurons; 23 ± 6.5 PSCs/neuron; Fig. 4*B*). The stimulation intensity was then increased to recruit additional axons (Fig. 4*B*, inset) until all accessible presynaptic axons were stimulated to yield the maximal evoked PSC amplitude (99.19 ± 74.45 pA; *n* = 60 neurons; 27.5 ± 8.5 PSCs/neuron; Fig. 4*B*, inset). The number of axons synapsing onto the MOC neuron was estimated for each cell using two methods. For all cells, we calculated the convergence ratio, the maximum divided by the minimum evoked PSC amplitude (Kim and Kandler, 2003, 2010; Noh et al., 2010). Convergence ratios ranged from 0.94 to 21.50 (3.64 ± 2.16; *n* = 60 neurons). For the subset of neurons in which we performed fine-grained increases in electrical stimulus intensity while measuring ePSC amplitude, we also determined the number of axons synapsing onto each MOC neuron using k-means clustering (Materials and Methods; Ferragamo et al., 1998), which is more sensitive than the convergence ratio to heterogeneous postsynaptic responses evoked from different presynaptic axons. The results of the two methods did not differ (CR, 2.35 ± 0.82; k-means clustering, 4.0 ± 1.5; Wilcoxon signed-rank test: W = 17, *n* = 8 neurons, *p* = 0.95). The number of MNTB neurons synapsing onto an MOC neuron determined here is likely an underestimate, as axons may be cut during the slicing procedure. Further, the wide range of values suggests a large variability in the number of inhibitory axons synapsing onto a single MOC neuron.

### Developmental changes in inhibitory inputs to the MOC

Brainstem auditory axons, including MNTB projections to other SOC nuclei such as the LSO, undergo dramatic functional and structural plasticity that occurs in stages throughout the first 3 postnatal weeks in rodents (Sanes and Siverls, 1991; Sanes and Friauf, 2000; Kim and Kandler, 2003; Werthat et al., 2008; Kandler et al., 2009; Clause et al., 2014). If presumed MNTB synapses onto MOC neurons were a result of excess axon projections that exceed the boundaries of the MSO or LSO but that would later be pruned, it is possible that inhibitory synapses from MNTB neurons onto MOC neurons are no longer present in the mature animal. To address this, we compared the strength and number of inhibitory inputs to MOC neurons evoked from MNTB bundle stimulation by age and determined that there is no change in the number of MNTB neurons synapsing onto MOC neurons, measured by convergence ratio, with age through P36 (Fig. 4*C*; linear regression: *r*^2^ = −0.01158, *p* = 0.95). MNTB synapses onto MOC neurons are maintained at ages well after the pruning of synaptic contacts onto other auditory neurons, suggesting that MNTB connections to MOC neurons are maintained in maturity and most likely originate from dedicated axon collaterals.

We then determined the neurotransmitters mediating PSCs evoked by electrical stimulation of axons near the MNTB. The intensity of the electrical stimulus was set to an intermediate value that reliably evoked PSCs (stimulation intensity, 280 ± 210 μA; rate of failures, 6.5% in 36 neurons; 5 μm CNQX in aCSF). With this range of stimulus intensities, ePSCs had amplitudes of 79.4 ± 57.1 pA and time constants of decay of 14.3 ± 4.4 ms (*n* = 22 neurons, 17–30 ePSCs/neuron). To test whether stimulated axons released GABA or glycine, postsynaptic receptors were blocked with strychnine or gabazine, either alone or in combination. Strychnine application significantly reduced the amplitude of ePSCs (CNQX control: 89.9 ± 68.3 pA; CNQX plus strychnine: 34.6 ± 19.9 pA; one-tailed Wilcoxon signed-rank test: W = 120, *n* = 15 neurons; 78–232 stimulations/neuron; *p* = 3.1E-5; Fig. 4*D*). Similar to sPSCs in strychnine, ePSCs had slightly longer time constants of decay, but the effect did not reach statistical significance (CNQX control: 18.3 ± 6.4 ms; CNQX plus strychnine: 20.9 ± 6.5 ms; paired Wilcoxon signed-rank test: W = 34, *n* = 15 neurons, 2–30 ePSCs/neuron, *p* = 0.15). Similarly, gabazine application also significantly reduced the amplitude of evoked PSCs (CNQX: 71.0 ± 49.7 pA; CNQX plus gabazine: 18.3 ± 7.6 pA; one-tailed Wilcoxon signed-rank test: W = 28, *n* = 7 neurons, 70–200 stimulations/neuron, *p* = 0.00781; Fig. 4*E*), with no effect on the kinetics of ePSCs (CNQX: 11.5 ± 1.9 ms; CNQX plus gabazine: 11.1 ± 3.8 ms; paired Wilcoxon signed-rank test: W = 8, *n* = 5 neurons, 2–30 ePSCs/neuron, *p* = 1), similar to the effects on sPSCs. Sequential application of strychnine and gabazine nearly eliminated ePSCs (CNQX plus strychnine: 24.8 ± 9.5 pA; CNQX plus strychnine plus gabazine: 11.6 ± 5.3 pA; *n* = 9 neurons; 70–200 stimulations/neuron; one-tailed Wilcoxon signed-rank test: W = 45, *p* = 0.00195; Fig. 4*F*). These results indicate that electrical stimulation of axons in and near the MNTB evokes both GABA and glycine release.

### MNTB neurons provide inhibitory synaptic inputs to MOC neurons

**Figure 5.**
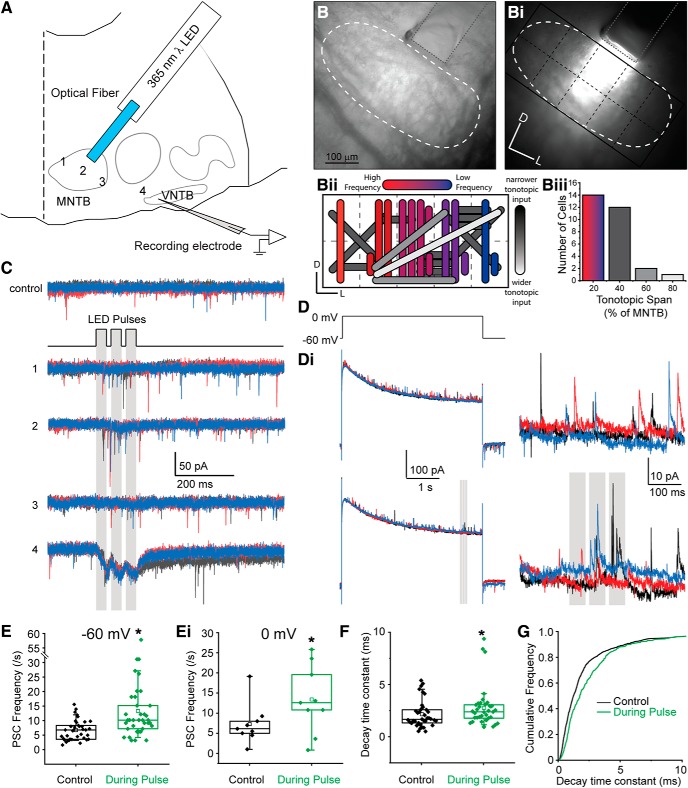
Glutamate uncaging activation of MNTB neurons evokes PSCs in MOC neurons. ***A***, Schematic of patch-clamp recording of MOC neurons and fiber-optic position in the MNTB for focal UV illumination for glutamate uncaging. Numbers represent the approximate location of illumination during the recordings shown in ***C***. ***B***, ***Bi***, Images of the LED fiber (gray dashed line) placement above the MNTB (white dashed outline). ***B***, DIC image of MNTB and optical fiber. ***Bi***, Fluorescence image of the LED illumination area during flash (405 nm epifluorescence filter) with approximate stimulation grid (black boxed region) superimposed above MNTB. ***Bii***, Diagram of activation area of MOC neurons. The lines connect the two optimal MNTB stimulation regions for each MOC cell. Line color indicates an approximation of tonotopic input areas with solid colored lines representing cells best stimulated along isofrequency regions and grayscale lines approximating tonotopic input breadth. Lines were placed to avoid overlap and do not represent the exact location of stimulation. ***Biii***, Quantification of cells represented in ***Bii*** suggests that the majority of MOC neurons receive narrow band inhibitory input from MNTB neurons. ***C***, Sets of three sweeps (each indicated by a different color) from a representative MOC neuron during an uncaging protocol for control (no illumination) and four discrete locations in the MNTB. Vertical gray bars indicate LED pulses. Illumination in location 2 yielded the most evoked events. Location 4 is shown for comparison and represents the response of uncaging near the MOC cell, activating glutamate receptors directly. ***D***, Voltage-clamp protocol used to isolate inhibitory PSCs. ***Di***, Left, Current response during the step protocol for control (top) and uncaging (bottom). Right, Expanded view of the trace during the approximate steady-state response for control (top) and uncaging (bottom) showing evoked PSCs during the uncaging light pulses (gray bars). ***E***, ***Ei***, Population data comparing the PSC rate for control and for uncaging at −60 mV (***E***; *n* = 37) and 0 mV (***Ei***; *n* = 9) holding potential. ***F***, Measures of decay kinetics of PSCs for control and during glutamate uncaging for each cell at −60 mV show a small but significant slowing of kinetics during evoked PSCs. ***G***, Cumulative frequency plot for the decay kinetics of individual PSCs in control (*n* = 1169) and during glutamate uncaging (*n* = 440) at −60 mV shows slower decay kinetics of PSCs evoked during the uncaging pulse. In all panels an asterisk indicates *p* < 0.05.

Electrical stimulation in or lateral to the MNTB will not only activate MNTB axons, but also nonselectively activate other axons of passage. To confirm the presence of direct MNTB synapses onto MOC neurons, we specifically activated MNTB somata using focal hydrolysis of “caged” MNI-caged-L-glutamate (0.2 mm), which is inert until exposed to ultraviolet light. “Uncaging” releases active glutamate in the illumination region to activate neurons via somatic and dendritic glutamate receptors. In proof-of-concept experiments, we recorded from an MNTB neuron and “uncaged” glutamate in the MNTB (3 × 50 ms pulses of light with 10 ms interpulse interval; 365 nm LED illumination transmitted via a 100-μm-diameter optical fiber; Fig. 5*A*, schematic). Glutamate uncaging evoked action potentials in P13–P16 MNTB neurons. At this age range, there was a diversity of spiking patterns (latency, 7.25 ± 3.57 ms; range, 3.38–38.2 ms, *n* = 8), with many MNTB neurons exhibiting multiple spikes in response to the three light pulses.

The presence of direct synapses between MNTB and MOC neurons was then tested during recordings from MOC neurons while glutamate was focally uncaged to stimulate MNTB neurons. The tissue orientation was changed in different experiments so that sometimes the optical fiber angled either dorsally (24 experiments) or ventrally (22 experiments). The illumination area for a ventrally oriented fiber is shown (Fig. 5*B*,*Bi*). MNTB activation via glutamate uncaging evoked PSCs in the postsynaptic MOC neuron in 27 of 46 experiments (arbitrary definition of an evoked response is a >50% increase in PSC frequency during stimulation; however, the population data below include all cells), comparable to the ∼60% of experiments in which direct electrical stimulation of presynaptic axons evoked PSCs (above). Responses at a −60 mV holding potential from a representative cell are shown in Figure 5*C* for positions indicated in Figure 5*A*. In this example MOC cell, glutamate uncaging at the medial edge of the ipsilateral MNTB evoked few PSCs (Fig. 5*C*, position 1). Uncaging near the center of the MNTB evoked the most robust response (Fig. 5*C*, position 2). Importantly, moving the optical fiber laterally and toward the MOC neuron resulted in fewer evoked PSCs (Fig. 5*C*, position 3), confirming that glutamate uncaging-evoked events were not the result of nonselective stimulation of MNTB axons or other fibers of passage. Direct glutamate uncaging over MOC dendrites evoked slow inward currents, with kinetics distinct from synaptic currents (Fig. 5*C*, position 4). Across the entire population of PSCs recorded using this protocol, we quantified the rate of PSCs occurring during the glutamate uncaging stimulus and compared this to the control (no light stimulus). We observed a significant increase in PSC rate during light stimulation (control PSC rate: 6.6 ± 2.8/s; during light pulse: 10.0 ± 4.0/s; paired-sample Wilcoxon signed-rank test: W = 36, *n* = 37 neurons, *p* = 1.0E-07; Fig. 5*C*,*E*). These data confirm that the activation of MNTB neurons via glutamate uncaging resulted in evoked PSCs in MOC neurons. To determine whether uncaging-evoked PSCs in MOC neurons were indeed inhibitory, we first measured the kinetics of evoked events relative to spontaneous PSCs. We compared the time constant of decay of PSCs for each cell in each condition and observed that PSCs evoked by glutamate uncaging had slightly longer time constants compared with control (control: 1.74 ± 0.61 ms; during light pulse: 2.45 ± 0.67 ms; paired-sample Wilcoxon signed-rank test: W = 190, *n* = 37 neurons, *p* = 0.014; Fig. 5*F*,*G*), which is consistent with the slower kinetics of pharmacologically isolated inhibitory synaptic responses (Fig. 3). The experiment was then repeated with the MOC neuron membrane potential held at 0 mV, the reversal potential of AMPA receptor currents, to isolate inhibitory PSCs. Glutamate uncaging significantly increased the rate of PSCs, which were outward at 0 mV (control: 6.1 ± 1.6/s; during light pulse: 12.6 ± 7.0/s; paired-sample Wilcoxon signed-rank test: W = 3, *n* = 9 neurons, *p* = 0.020; Fig. 5*Ei*). The latency to the first evoked PSC was also measured (−60 mV: 56.14 ± 20.08 ms; *n* = 24; 0 mV: 58.72 ± 19.23 ms; *n* = 9) and was similar at both holding potentials (Mann–Whitney test: U = 130, *p* = 0.38). These data confirm that MNTB neurons form inhibitory synapses onto MOC neurons. A rough, qualitative assessment of the “activation area” for a given MOC cell was performed by comparing the location of the two optimal stimulation sites (Fig. 5*Bi*, approximate grid). In 29 experiments, 14 neurons were best stimulated along isofrequency regions (*n* = 5 that could only be stimulated in one region; *n* = 9 that were stimulated in vertically adjacent areas). An additional 12 neurons could be stimulated in two horizontally adjacent areas (spanning two frequency areas). Only three neurons tested had stimulation areas spanning across nonadjacent areas (Fig. 5*Bii*,*Biii*). This suggests that the majority of MOC neurons may be innervated by MNTB neurons within an isofrequency band, similar to other MNTB-SOC nuclei innervation patterns.

### Sustained activation of presynaptic terminals

**Figure 6.**
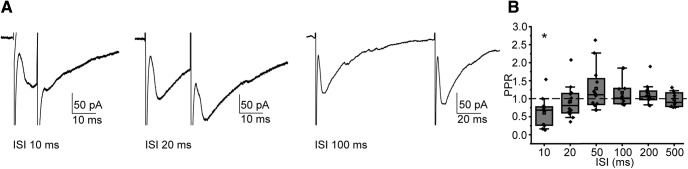
MNTB synapses onto MOC neurons depress. ***A***, Example voltage-clamp traces of ePSCs evoked from electrical stimulation of presynaptic axons, stimulated as in Figure 4*A*. Axons were stimulated twice at the ISIs indicated. ***B***, Summary data of PPRs for 12 neurons at the ISI indicated. Asterisk indicates *p* < 0.05.

**Figure 7.**
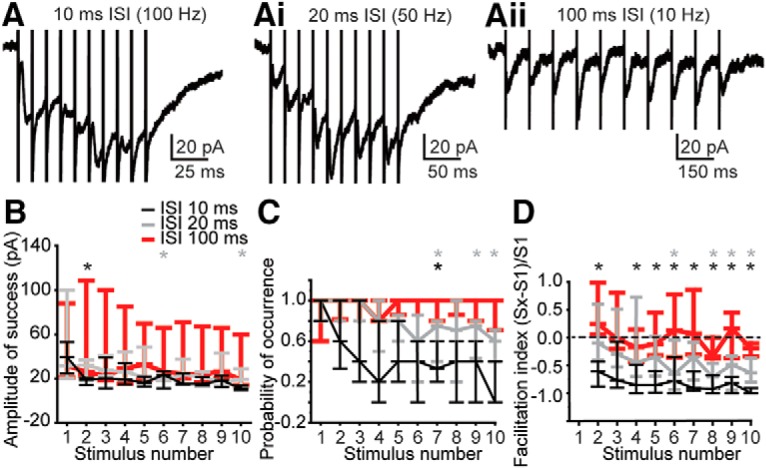
MNTB synapses onto MOC neurons depress when stimulated repetitively. ***A–Aii***, Example voltage-clamp traces from the same MOC neuron while electrically stimulating presynaptic axons (as in Fig. 4*A*) using trains of pulses at the ISI indicated. ***B***, Plot of amplitude of successful ePSCs during trains of MNTB axon stimulation at 100 Hz (black, 10 ms ISI), 50 Hz (gray, 20 ms ISI), and 10 Hz (red, 100 ms ISI); *n* = 7 neurons. ***C***, Probability of recording an ePSC during a train of MNTB stimulation, using the same data as in ***B***. ***D***, Facilitation index [(Sx − S1)/S1] for data in ***B*** and ***C***. Index <0 indicates synaptic depression, and >0 indicates facilitation. ***B–D***, plots represent median and quartile ranges. In all panels an asterisk indicates *p* < 0.05.

*In vitro*, MNTB neurons spike at the onset of a sustained depolarization, but will spike repetitively in response to trains of stimulation (Banks and Smith, 1992; Wu and Kelly, 1993; Forsythe, 1994; Barnes-Davies and Forsythe, 1995; Brew and Forsythe, 1995; Taschenberger and von Gersdorff, 2000; Futai et al., 2001; Joshi et al., 2004; Klug and Trussell, 2006; Hermann et al., 2007) or in phase with sound stimuli *in vivo* (Smith et al., 1998; Kopp-Scheinpflug et al., 2003, 2008; Tolnai et al., 2008). As a first step in characterizing the effect of repetitive MNTB activity on the inhibition of MOC neurons, we electrically stimulated MNTB axons in pairs and in trains at varying interstimulus intervals (ISIs; Fig. 6*A*). In paired-pulse experiments at an ISI of 10 ms, the evoked postsynaptic responses to the second pulse were significantly smaller than the responses to the first pulse, as determined by calculation of the paired-pulse ratio (PPR; 10 ms ISI PPR: 0.68 ± 0.36; two-tailed Wilcoxon signed-rank test: W = 8, *n* = 12 neurons, *p* = 0.012; Fig. 6*B*), indicating synaptic depression. The extracellular calcium concentration can affect the magnitude of depression by affecting presynaptic release probability, so we also tested PPR in 1.2 mm extracellular calcium and found PPR to be unchanged (10 ms ISI PPR in 1.2 mm Ca^2+^: 0.54 ± 0.12; Mann–Whitney test vs 2.0 mm Ca^2+^: U = 69, *p* = 0.64). The lack of change in PPR with a lower calcium concentration suggests that at the MNTB–MOC synapse, both presynaptic and postsynaptic mechanisms are likely to be involved in synaptic depression. PPRs were not significantly different from zero at the longer intervals tested from 20 to 500 ms (Fig. 6*B*). We then electrically stimulated MNTB axons using trains of pulses to more closely imitate presynaptic activity in response to sound stimuli (Fig. 7*A–Aii*). Axons were stimulated with trains of 10 pulses, at an intermediate stimulation intensity yielding reliable ePSCs to single pulses (stimulation current average ± SD, 341.71 ± 225.20 μA; range, 60–640 μA; 5–7 sweeps/stimulation rate; 5 μm CNQX included in aCSF). We calculated the amplitude of successful PSCs (A; Fig. 7*B*), and the probability of PSC occurrence (P; Fig. 7*C*) at each stimulus, then computed the facilitation index [x = stimulus number, Sx = Ax * Px, index = (Sx − S1)/S1; Fig. 7*D*; Goutman et al., 2005; Ballestero et al., 2011]. A negative facilitation index indicates synaptic depression. As in paired-pulse experiments, ePSCs were significantly depressed at MNTB axon stimulation rates of 100 Hz (ISI, 10 ms) beginning with the second pulse (one-tailed Wilcoxon signed-rank test: test index ≠ 0; *n* = 7 neurons; *p* < 0.05). Similar to paired-pulse data, synaptic depression did not occur when axons were stimulated at rates of 50 Hz (ISI, 20 ms) for pulses two to five, but then synaptic depression was evident beginning at the sixth pulse in the train. The depression of MNTB–MOC synapses during repetitive stimulation of MNTB axons suggests that during a sustained sound, MNTB inhibition of MOC neurons may decrease, thereby allowing increased activity in the MOC neuron and enhanced MOC-mediated inhibition of cochlear OHC activity.

### Inhibition of MOC neurons

**Figure 8.**
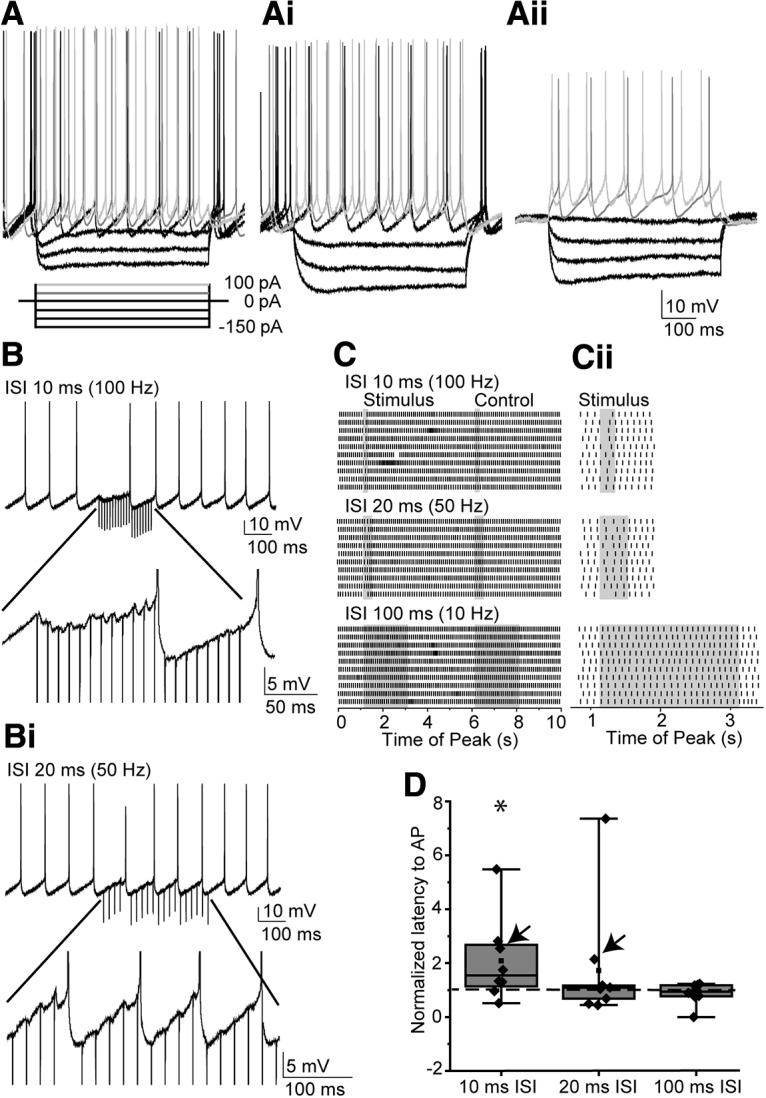
Synaptic inputs from the MNTB inhibit action potentials in MOC neurons. ***A–Aii***, Current-clamp traces from three representative MOC neurons. Current step protocol under ***A*** applies to all panels. ***B***, Example current-clamp traces from a representative neuron (indicated by arrows in ***D***) of spontaneous action potentials, and the effect of 100 Hz MNTB axon stimulation (downward lines are the MNTB–axon stimulation artifact). Bottom, Zoom of IPSPs. ***Bi***, Current-clamp recording from the same neuron as in ***B***, MNTB axon stimulation at 50 Hz. Bottom, Zoom of IPSPs from ***Bi***. ***C***, Raster plots of action potentials in 10 traces per stimulation rate, same neuron as in ***B*** and ***Bi***. Shaded area indicates the region of MNTB axon stimulation (20 pulses) or the control region 5 s after the start of stimulation. ***Ci***, Zoom of stimulation windows. ***D***, Summary data for 8–9 MOC neurons of action potential latency after the start of MNTB axon stimulation, normalized to control window. Individual neuron data are overlaid. Arrows indicate neurons selected for ***B*** and ***C***. Asterisk indicates *p* < 0.05.

Finally, we tested the ability of synaptic inputs from MNTB neurons to inhibit activity in MOC neurons. In current-clamp at physiological temperature, MOC neurons had spontaneous action potentials (rate, 7.3 ± 2.53/s; *n* = 9 neurons; Fig. 8*A*,*B*) at resting membrane potential. There was no relationship between age and action potential rate (age, 16 ± 2 d; age range, P14–P23; *p* = 0.8). Electrical stimulation of MNTB axons (as above) was performed while recording in voltage-clamp to find a stimulus intensity that reliably evoked IPSCs (in 5 μm CNQX). Then, in current-clamp recording configuration, the magnitude of hyperpolarizing postsynaptic potentials evoked by single stimulating pulses was measured (−0.64 ± 0.21 mV; *n* = 9 neurons, including two cells that did not have measurable IPSPs). MNTB axon stimulation was applied in trains of 20 pulses at rates of 10, 50, and 100 Hz (Fig. 8*B*,*Bi*, example traces for 50 and 100 Hz). The impact of evoked IPSPs on spontaneous action potentials was assessed by comparing first spike latency during evoked IPSPs to that during an identical time window without stimulation. Stimulus windows alternated with control windows with an interval of 5 s separating the start of each window (Fig. 8*C*,*Ci*, shaded regions indicate stimulus or control windows). IPSPs increased the latency to action potentials in the stimulus time window relative to the latency to action potential in the control window when stimulated at a rate of 100 Hz (ISI, 10 ms), but not 50 or 10 Hz (100 Hz rate normalized relative to control: 1.54 ± 0.79; one-sample Wilcoxon signed-rank test: W = 31, *n* = 8 neurons, 20 trains/neuron, *p* = 0.039, 50 Hz rate normalized relative to control: 1.09 ± 0.41; *p* = 0.33; 10 Hz rate normalized relative to control: 0.94 ± 0.21; *p* = 0.54; Fig. 8*D*) and, occasionally, completely suppressed action potentials for the duration of the train. If the delay of action potentials by IPSPs in MOC neurons observed here also occurs *in vivo*, this indicates that MNTB neurons can inhibit MOC activity, which would in turn delay MOC synaptic suppression of cochlear OHCs.

## Discussion

The synaptic circuitry regulating MOC efferent neurons has been poorly understood due to difficulties locating the somata for *in vitro* experiments. We overcame this limitation by genetically labeling MOC neurons to distinguish them from surrounding neurons, including those that form GABAergic and glycinergic synapses onto MNTB neurons (Albrecht et al., 2014), and other less well defined neurons with distinct electrophysiological properties, neurotransmitter labeling, and morphology (Helfert et al., 1989; Vetter et al., 1991; Robertson, 1996). The few patch-clamp electrophysiology reports from MOC neurons located via retrograde label from the cochlea or identified *post hoc* using immunolabels demonstrated A-type potassium channels and repetitive spiking in MOC neurons (Robertson, 1996; Fujino et al., 1997; Tong et al., 2013). Patch-clamp recordings with *post hoc* sorting of VNTB cells by morphology suggests that putative MOC neurons have both excitatory and inhibitory synaptic inputs, but presynaptic cells or neurotransmitters were not determined (Robertson, 1996). Here we use patch-clamp electrophysiology recordings of spontaneous or evoked PSCs to confirm GABAergic and glycinergic synapses onto identified MOC neurons. Further, we identify MNTB neurons as a source of inhibition, demonstrating the function of a previously unknown MNTB–MOC circuit likely driven by sound to the same ear as dominant excitatory inputs, based upon MNTB innervation (Morest, 1968; Warr, 1972; Friauf and Ostwald, 1988; Spirou et al., 1990; Kuwabara et al., 1991; Smith et al., 1991). MNTB synapses onto MOC neurons can inhibit spontaneous action potentials, but depress with repeat stimulation, suggesting that if MNTB inhibition of MOC neurons functions *in vivo*, as has been shown in this *in vitro* preparation, the synaptic inhibition may have the greatest influence at sound onset.

### Convergence of synaptic excitation and inhibition

The role of MNTB inhibition of MOC neurons will depend on inhibitory synapse efficacy, given the intrinsic MOC properties and integration with excitatory synaptic activity. Sound-evoked excitation is likely via VCN T-stellate/multipolar planar neurons in a “sound reflex” pathway, primarily contralateral to MOC somata (Brown et al., 2003; de Venecia et al., 2005; Darrow et al., 2012). T-stellate cells have sharp tuning curves and respond to acoustic stimuli with “chopper” type sustained responses and linear increases in action potential rate up to 700 Hz with increased sound intensity (Rhode et al., 1983; Rouiller and Ryugo, 1984; Rhode and Smith, 1986; Blackburn and Sachs, 1989; Smith and Rhode, 1989; Palmer et al., 1996, 2003). MOC neurons are also choppers, but rarely exceed 100 Hz firing (Fex, 1962a; Robertson and Gummer, 1985; Liberman and Brown, 1986; Brown, 1989), suggesting that MOC action potential rates are limited by intrinsic electrical properties, ineffective synaptic drive from T-stellate cells, or inhibition. MNTB inhibition of MOC neurons is driven from the globular bushy cells (GBCs) of the contralateral VCN. The GBCs have primary-like, occasionally with notch, sound responses. GBCs spike at high rates at sound onset followed by a decreased spike rate, and change spike rate over a narrow intensity range (Brownell, 1975; Rhode et al., 1983; Rhode and Smith, 1986; Smith and Rhode, 1987; Young et al., 1988; Spirou et al., 1990; Smith et al., 1991; Joris et al., 1994; Rhode, 2008). GBCs have specializations for high-fidelity transmission, including large, myelinated axons, and terminations on MNTB neurons in the Calyx of Held (Held, 1893; Ramón y Cajal, 1909; Warr, 1972; Spirou et al., 1990; Kuwabara et al., 1991; Smith et al., 1991; Forsythe, 1994; Ford et al., 2015; Stange-Marten et al., 2017), a famously powerful and high-fidelity synaptic terminal (Guinan and Li, 1990; Wu and Kelly, 1993; Barnes-Davies and Forsythe, 1995; Borst et al., 1995; Brew and Forsythe, 1995; Chuhma and Ohmori, 1998; Taschenberger and von Gersdorff, 2000; Futai et al., 2001; but see Kopp-Scheinpflug et al., 2003; Klug and Trussell, 2006; Hermann et al., 2007; McLaughlin et al., 2014), yielding similar “primary-like with notch” response patterns in MNTB neurons (Guinan and Li, 1990; Smith et al., 1998; Paolini et al., 2001; Kopp-Scheinpflug et al., 2008; Tolnai et al., 2008).

MOC activity patterns *in vivo* likely depend on the convergence of an excitatory pathway via T-stellate cells and an inhibitory pathway via GBC and MNTB neurons, although other modulating synapses may play a role. Given that the GBC–MNTB pathway consists of exceptionally fast and specialized axons and synapses, inhibition could arrive synchronously with or earlier than excitation (Brand et al., 2002; Pecka et al., 2008; Roberts et al., 2013; Beiderbeck et al., 2018). We predict that GBC-MNTB inhibition likely dominates at sound onset and then decays, while sustained T-stellate excitation may have a greater effect at higher sound intensities and throughout the sound duration. In addition, synaptic depression at MNTB–MOC synapses demonstrated here suggests that MNTB inhibition is strongest at stimulus onset. Indeed, inhibition from the MNTB delayed MOC action potentials but rarely suppressed them entirely. Combined, integration of excitatory and inhibitory synapses could result in MOC action potential failure at sound onset of any intensity due to rapid initial inhibition from MNTB neurons, followed by regular MOC spiking driven by T-stellate inputs that increase activity with sound intensity. Thus, strong MNTB inhibition may delay, but not completely suppress, MOC cochlear effects.

### Strength of MNTB–MOC synapses

Both electrical stimulation of MNTB axons and glutamate uncaging evoked PSCs in ∼60% of MOC neurons. The lack of MNTB–MOC responses in some experiments could be an artifact of severed input axons or MOC dendrites in slices, or it could indicate MNTB innervation of a subset of MOC neurons. Indeed, a survey of published MNTB neuron morphology reports suggests axon collaterals to the VNTB, but with a smaller innervation area than to other SOC nuclei (Morest, 1968; Kuwabara and Zook, 1991, 1992; Kuwabara et al., 1991; Banks and Smith, 1992; Smith et al., 1998). In addition, the latency to glutamate uncaging-evoked PSCs in MOC neurons was longer than the latency to action potentials evoked in MNTB neurons. The long latency could be due to disynaptic or gap junction-mediated activation involving an additional cell. However, it is likely an artifact of the variable activation of MNTB neurons using glutamate uncaging, due to factors including MNTB neuron depth in the slice, with deeper MNTB somata being weakly activated due to light scattering, or asynchronous MNTB activation. The variable latency could also be due to occasional failures of action potential propagation into the axon collateral to the VNTB, as observed in other neurons (Deschênes and Landry, 1980; Debanne et al., 1997, 2011; Bucher and Goaillard, 2011). We also cannot exclude that electrical axon stimulation evokes inhibition from other cells. Inhibition does suppress MOC activity in our preparation and likely shapes MOC sound-driven activity. Perhaps *in vivo*, the high spontaneous rate of MNTB activity (Smith et al., 1998; Kopp-Scheinpflug et al., 2003) provides tonic inhibition of MOC neurons, which are rarely spontaneously active (Robertson and Gummer, 1985; Liberman and Brown, 1986; Brown, 1989). However, determination of the full spectrum of the effect of *in vivo* MNTB–MOC inhibition awaits additional experimentation.

### Effect of MNTB inhibition of MOC neurons on cochlear responses

MOC neuron cochlear activity in mature animals is determined by action potentials that propagate along the axon to synapses onto OHCs (Spoendlin, 1969; Warr and Guinan, 1979; Mountain, 1980; Siegel and Kim, 1982; Brown, 1987; Maison et al., 2003). The specialized OHC feature of electromotility is implicated in lowering auditory thresholds at characteristic frequencies and sharpening tuning curves, improving signal detection (Brownell et al., 1985; Evans and Dallos, 1993). By hyperpolarizing OHCs via cholinergic receptor activation of potassium channels to suppress OHC function (Elgoyhen et al., 1994, 2001; Dulon et al., 1998; Vetter et al., 1999; Glowatzki and Fuchs, 2000; Oliver et al., 2000; Weisstaub et al., 2002; Nie et al., 2004; Goutman et al., 2005; Wersinger et al., 2010), direct electrical activation of MOC axons raises auditory thresholds, reduces auditory nerve action potential rates, suppresses otoacoustic emissions, and broadens auditory nerve tuning curves (Galambos, 1956; Fex, 1962b; Wiederhold and Kiang, 1970; Mountain, 1980; Siegel and Kim, 1982; Art et al., 1985). Direct electrical MOC activation evoked the largest auditory nerve compound action potential “level shifts” at high stimulation rates of 200–400 Hz (Galambos, 1956; Guinan and Gifford, 1988; Guinan, 1996). However, sound-driven action potential rates *in vivo* in MOC axons have not been measured above 120 Hz, with an average rate of ∼50 Hz (Robertson and Gummer, 1985; Liberman and Brown, 1986; Brown, 1989), and there is a linear relationship between MOC action potential rates and cochlear suppression of auditory nerve activity up to ∼100 Hz of MOC spiking (Galambos, 1956; Guinan and Gifford, 1988; Ballestero et al., 2011). T-stellate neurons have higher maximum action potential rates in response to sound compared with MOC neurons, implicating a mechanism that prevents 1:1 correspondence between T-stellate and MOC action potential rates and prevents MOC neurons from spiking at their maximal effective rate during intermediate sound intensities. The efficacy of the synaptic transfer of T-stellate to MOC neurons is unknown and is possibly reduced by presynaptic mechanisms. However, MNTB inhibition of the MOC is another potential mechanism of limiting MOC neuron activation, to reduce MOC action potential rates to within the linear range of their cochlear activity and also to delay cochlear activity.
